# (Chlorido/bromido)[(1,2,5,6-η)-cyclo­octa-1,5-diene](4-isopropyl-1-methyl-1,2,4-triazol-5-yl­idene)rhodium(I)

**DOI:** 10.1107/S2414314621008117

**Published:** 2021-08-13

**Authors:** Joshua Rushlow, Andrei V. Astashkin, Daniel R. Albert, Edward Rajaseelan

**Affiliations:** a Department of Chemistry, Millersville University, Millersville, PA 17551, USA; b Department of Chemistry and Biochemistry, The University of Arizona, Tuscon, AZ, 85716, USA; Vienna University of Technology, Austria

**Keywords:** crystal structure, rhodium, N-heterocyclic carbenes, Cl/Br substitutional disorder

## Abstract

A new triazole-based neutral Rh^I^ complex, [Rh(Cl_0.846_Br_0.154_)(C_6_H_11_N_3_)(C_8_H_12_)], has been synthesized and structurally characterized. The Rh^I^ atom has a distorted square-planar coordination environment, formed by a bidentate cyclo­octa-1,5-diene (COD) ligand, an N-heterocyclic carbene and a halide ligand that shows substitutional disorder (Cl:Br = 0.846:0.154). No significant inter­molecular inter­actions other than van der Waals forces are found in the crystal structure. Diffraction data indicated a two-component inversion twin with a ratio of 0.95 (5):0.05 (5).

## Abstract 2

The Rh^I^ atom in the title compound has a distorted square-planar coordination environment, defined by a bidentate cyclo­octa-1,5-diene (COD) ligand, an N-heterocyclic carbene and a chloride ligand with bromide substitutional disorder.

## Graphical abstract

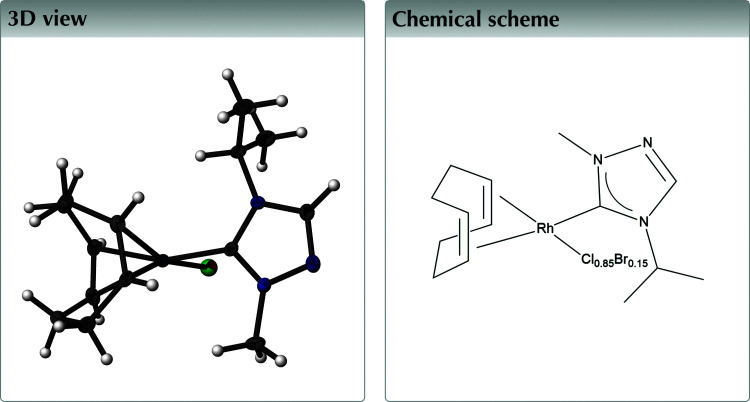

## Structure description

Transition-metal complexes containing N-heterocyclic carbene (NHC) ligands have been studied extensively in homogeneous catalysis (Díez-Gonzáles *et al.*, 2009 ▸), especially in transfer hydrogenation of unsaturated bonds (Ruff *et al.*, 2016 ▸; Zuo *et al.*, 2014 ▸). The NHC ligands can be tuned sterically and electronically by having different substituents on the nitro­gen atoms (Gusev, 2009 ▸). Many imidazole- and triazole-based NHC rhodium and iridium complexes have been synthesized and structurally characterized (Herrmann *et al.*, 2006 ▸; Wang & Lin, 1998 ▸; Chianese *et al.*, 2004 ▸; Nichol *et al.*, 2009 ▸, 2010 ▸, 2011 ▸, 2012 ▸; Idrees *et al.*, 2017*a* ▸,*b* ▸; Rood *et al.*, 2021 ▸). Their catalytic activities in the transfer hydrogenation of ketones and imines have also been studied and reported (Hillier *et al.*, 2001 ▸; Albrecht *et al.*, 2002 ▸; Gnanamgari *et al.*, 2007 ▸).

**Figure 1:**
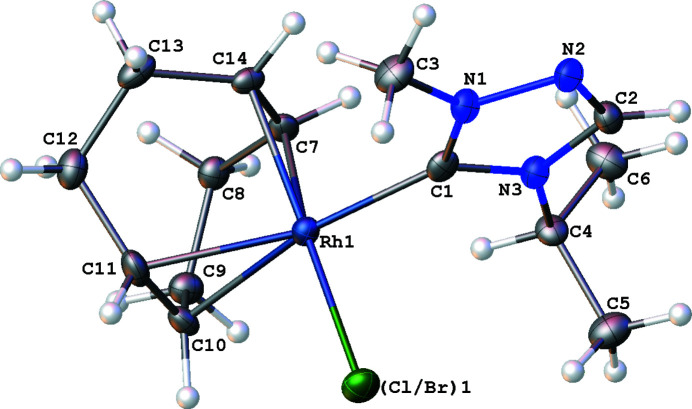
The mol­ecular structure of the title compound (**3**) with displacement ellipsoids drawn at the 50% probability level.

**Figure 2:**
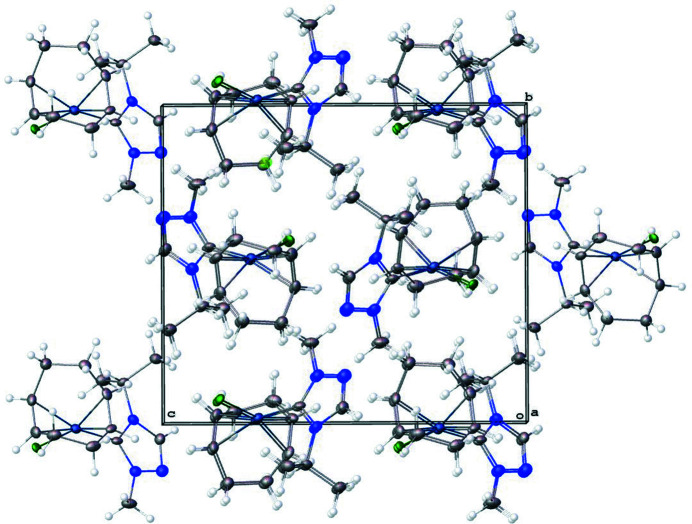
Crystal packing diagram of the title compound (**3**) along the *a* axis.

The mol­ecular structure of the title complex, [Rh(Cl_0.846_Br_0.154_)(C_6_H_11_N_3_)(C_8_H_12_)] (**3**), is illustrated in Fig. 1 ▸. The coordination environment around the Rh^I^ ion, formed by the bidentate cyclo­octa-1,5-diene (COD), NHC, and halide (Cl,Br) ligands is distorted square-planar. The Rh—C(NHC) bond length is found to be 2.016 (5) Å. The C(NHC)—Rh—(Cl,Br) bond angle is 87.93 (14)°. The N—(carbene)—N bond angle in the triazole-based carbene is 103.1 (4)°. Fig. 2 ▸ shows the crystal packing diagram of the complex. No non-covalent inter­actions exist between atoms that are closer than the sum of the van der Waals radii.

**Figure 3:**
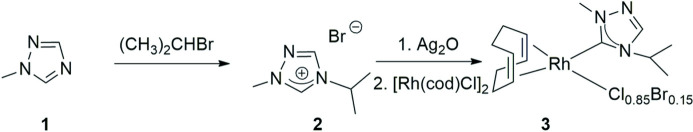
Reaction scheme summarizing the synthesis of the N-heterocyclic carbene ligand (**2**) and metal complex (**3**).

The rhodium–halide bond length in the reported structure is 2.4308 (11) Å, which is longer than previously reported Rh—Cl bond lengths, *viz*. 2.36–2.42 Å (Skelton *et al.*, 2019 ▸, 2020 ▸; Kalidasan *et al.*, 2015 ▸), and shorter than previously reported Rh—Br bond lengths, *viz* 2.49–2.55 Å (Benaissa *et al.*, 2017 ▸; Aznarez *et al.*, 2018 ▸), consistent with a Cl/Br substitutional disorder. The substitutional bromide likely comes from the triazolium salt (**2**) in the synthesis (Fig. 3 ▸).

## Synthesis and crystallization

1-Methyl triazole (**1**) was purchased from Matrix Scientific and the subsequent syntheses, as shown in Fig. 3 ▸, were performed using reagent-grade solvents without further purification. NMR spectra were recorded at room temperature in CDCl_3_ on a 400 MHz Varian spectrometer and referenced to the residual solvent peak (δ in ppm and *J* in Hz). The triazolium salt (**2**) was prepared by reacting (**1**) with isopropyl (*i*-Pr) bromide in toluene at reflux for 24 h followed by isolation with diethyl ether. The title metal complex (**3**) was synthesized by *in situ* transmetallation from the silver carbene complex of (**2**) (Chianese *et al.*, 2003 ▸). The pale-yellow complex (**3**) was obtained in qu­anti­tative yield.^1^H NMR: δ 7.89 (s, 1 H, N—C_3_H—N), 5.67 (*m*, 1 H, CH of *i*-Pr), 5.12 (*m*, 4 H, CH of COD), 4.34 (*s*, 3 H, CH_3_-N), 2.42–2.01 (*m*, 4 H, CH_2_ of COD), 1.57 (*m*, 6 H, CH_3_ of *i*-Pr). ^13^C NMR: 184.99 (*d*, Rh—C, *J*_C-Rh_ = 50.9), 139.07 (N—C_3_H—N), 99.80, 99.73, 99.29, 99.22 (CH of COD), 51.44 (CH_3_—N), 39.79 (CH of *i*-Pr), 33.08, 32.67, 29.01, 28.63 (CH_2_ of COD), 24.27, 23.34 (CH_3_ of *i*-Pr). Pale-yellow X-ray quality crystals of (**3**) were grown from 1:1, CH_2_Cl_2_/pentane by slow diffusion.

## Refinement

Crystal data, data collection and structure refinement details are summarized in Table 1 ▸. Using only the Cl ligand in the refinement did not account for all electron density at the ligand location, and therefore, a Cl/Br substitutional disorder was introduced for this site. The refinement was stabilized by forcing Cl and Br to have the same atomic coordinates and ADPs, using EXYZ and EADP instructions, respectively, in *SHELXL* (Sheldrick, 2015*b* ▸). The resulting occupancies for Cl and Br were about 85% and 15%, respectively. The crystal was refined as a two-component inversion twin with a ratio of 0.95 (5) to 0.05 (5).

## Supplementary Material

Crystal structure: contains datablock(s) I. DOI: 10.1107/S2414314621008117/wm4151sup1.cif

Structure factors: contains datablock(s) I. DOI: 10.1107/S2414314621008117/wm4151Isup2.hkl

CCDC reference: 2101889

Additional supporting information:  crystallographic information; 3D view; checkCIF report

## Figures and Tables

**Table 1:** Experimental details

Crystal data	
Chemical formula	[Rh(Br_0.154_Cl_0.846_)(C_6_H_11_N_3_)(C_8_H_12_)]
*M*_r_	378.60
Crystal system, space group	Orthorhombic, *P*2_1_2_1_2_1_
Temperature (K)	100
*a*, *b*, *c* (Å)	9.4146 (13), 11.9706 (17), 13.702 (2)
*V* (Å^3^)	1544.2 (4)
*Z*	4
Radiation type	Mo *K*α
μ (mm^−1^)	1.64
Crystal size (mm)	0.15 × 0.12 × 0.03

Data collection	
Diffractometer	Bruker APEXII CCD
Absorption correction	Multi-scan (*SADABS*; Krause *et al.*, 2015 ▸)
*T*_min_, *T*_max_	0.664, 0.745
No. of measured, independent and observed [*I* > 2σ(*I*)] reflections	16824, 3254, 3015
*R*_int_	0.057
(sin θ/λ)_max_ (Å^−1^)	0.632

Refinement	
*R*[*F* ^2^ > 2σ(*F* ^2^)], *wR*(*F* ^2^), *S*	0.027, 0.057, 1.03
No. of reflections	3254
No. of parameters	177
H-atom treatment	H-atom parameters constrained
Δρ_max_, Δρ_min_ (e Å^−3^)	0.65, −0.42
Absolute structure	Refined as an inversion twin.
Absolute structure parameter	0.05 (5)

Computer programs: *SAINT* (Bruker, 2007 ▸), *APEX2* (Bruker, 2007 ▸), *SHELXT* (Sheldrick, 2015*a* ▸), *SHELXL* (Sheldrick, 2015*b* ▸), and *OLEX2* (Dolomanov *et al.*, 2009 ▸).

## full crystallographic data

### Crystal data

**Table:**

[Rh(Br_0.154_Cl_0.846_)(C_6_H_11_N_3_)(C_8_H_12_)]	*D*_x_ = 1.628 Mg m^−^^3^
*M**_r_* = 378.60	Mo *K*α radiation, λ = 0.71073 Å
Orthorhombic, *P*2_1_2_1_2_1_	Cell parameters from 3850 reflections
*a* = 9.4146 (13) Å	θ = 2.6–25.5°
*b* = 11.9706 (17) Å	µ = 1.64 mm^−^^1^
*c* = 13.702 (2) Å	*T* = 100 K
*V* = 1544.2 (4) Å^3^	Plate, clear light colourless
*Z* = 4	0.15 × 0.12 × 0.03 mm
*F*(000) = 771	

### Data collection

**Table:**

Bruker APEXII CCD diffractometer	3015 reflections with *I* > 2σ(*I*)
φ and ω scans	*R*_int_ = 0.057
Absorption correction: multi-scan (*SADABS*; Krause *et al*., 2015)	θ_max_ = 26.7°, θ_min_ = 2.3°
*T*_min_ = 0.664, *T*_max_ = 0.745	*h* = −11→11
16824 measured reflections	*k* = −15→15
3254 independent reflections	*l* = −17→17

### Refinement

**Table:**

Refinement on *F*^2^	Hydrogen site location: inferred from neighbouring sites
Least-squares matrix: full	H-atom parameters constrained
*R*[*F*^2^ > 2σ(*F*^2^)] = 0.027	*w* = 1/[σ^2^(*F*_o_^2^) + (0.0271*P*)^2^ + 0.176*P*] where *P* = (*F*_o_^2^ + 2*F*_c_^2^)/3
*wR*(*F*^2^) = 0.057	(Δ/σ)_max_ < 0.001
*S* = 1.03	Δρ_max_ = 0.65 e Å^−^^3^
3254 reflections	Δρ_min_ = −0.42 e Å^−^^3^
177 parameters	Absolute structure: Refined as an inversion twin.
0 restraints	Absolute structure parameter: 0.05 (5)
Primary atom site location: dual	

### Special details

**Table:**

Geometry. All esds (except the esd in the dihedral angle between two l.s. planes) are estimated using the full covariance matrix. The cell esds are taken into account individually in the estimation of esds in distances, angles and torsion angles; correlations between esds in cell parameters are only used when they are defined by crystal symmetry. An approximate (isotropic) treatment of cell esds is used for estimating esds involving l.s. planes.
Refinement. Refined as a 2-component inversion twin.

### Fractional atomic coordinates and isotropic or equivalent isotropic displacement parameters (Å^2^)

**Table:**

	*x*	*y*	*z*	*U*_iso_*/*U*_eq_	Occ. (<1)
Rh1	0.36461 (3)	0.48473 (3)	0.25898 (2)	0.01379 (10)	
Cl1	0.55744 (11)	0.42693 (9)	0.15236 (8)	0.0233 (4)	0.846 (4)
N3	0.5884 (4)	0.5139 (3)	0.4182 (3)	0.0158 (8)	
N1	0.5001 (4)	0.3511 (3)	0.4233 (3)	0.0166 (9)	
N2	0.6009 (4)	0.3527 (3)	0.4974 (3)	0.0183 (9)	
C1	0.4910 (5)	0.4478 (4)	0.3733 (3)	0.0158 (10)	
C7	0.2352 (5)	0.5923 (5)	0.3445 (3)	0.0173 (11)	
H7	0.2842	0.6223	0.4036	0.021*	
C14	0.1882 (5)	0.4807 (5)	0.3551 (3)	0.0152 (10)	
H14	0.2100	0.4475	0.4204	0.018*	
C11	0.1905 (5)	0.4756 (6)	0.1477 (3)	0.0215 (12)	
H11	0.2079	0.4212	0.0936	0.026*	
C6	0.6157 (6)	0.7057 (4)	0.4812 (4)	0.0242 (11)	
H6A	0.5236	0.6976	0.5138	0.036*	
H6B	0.6295	0.7839	0.4621	0.036*	
H6C	0.6917	0.6831	0.5259	0.036*	
C13	0.0626 (6)	0.4277 (5)	0.3048 (4)	0.0213 (11)	
H13A	−0.0251	0.4480	0.3405	0.026*	
H13B	0.0727	0.3454	0.3077	0.026*	
C12	0.0468 (5)	0.4631 (4)	0.1982 (4)	0.0220 (12)	
H12A	−0.0105	0.4066	0.1630	0.026*	
H12B	−0.0047	0.5351	0.1952	0.026*	
C2	0.6523 (5)	0.4534 (4)	0.4915 (3)	0.0178 (10)	
H2	0.7250	0.4817	0.5327	0.021*	
C5	0.7597 (5)	0.6392 (4)	0.3365 (4)	0.0264 (12)	
H5A	0.8372	0.6178	0.3805	0.040*	
H5B	0.7747	0.7159	0.3138	0.040*	
H5C	0.7579	0.5884	0.2804	0.040*	
C8	0.1612 (5)	0.6824 (4)	0.2836 (3)	0.0178 (10)	
H8A	0.1784	0.7560	0.3144	0.021*	
H8B	0.0575	0.6685	0.2850	0.021*	
C4	0.6193 (5)	0.6320 (4)	0.3907 (3)	0.0172 (10)	
H4	0.5426	0.6579	0.3455	0.021*	
C10	0.2598 (5)	0.5757 (5)	0.1375 (3)	0.0174 (11)	
H10	0.3189	0.5812	0.0770	0.021*	
C9	0.2102 (5)	0.6876 (4)	0.1767 (4)	0.0203 (11)	
H9A	0.1306	0.7149	0.1359	0.024*	
H9B	0.2888	0.7421	0.1711	0.024*	
C3	0.4187 (6)	0.2494 (4)	0.4057 (4)	0.0235 (11)	
H3A	0.4571	0.2107	0.3485	0.035*	
H3B	0.3190	0.2687	0.3939	0.035*	
H3C	0.4253	0.2005	0.4629	0.035*	
Br1	0.55744 (11)	0.42693 (9)	0.15236 (8)	0.0233 (4)	0.154 (4)

### Atomic displacement parameters (Å^2^)

**Table:**

	*U*^11^	*U*^22^	*U*^33^	*U*^12^	*U*^13^	*U*^23^
Rh1	0.01309 (16)	0.01269 (16)	0.01558 (16)	0.00094 (13)	0.00034 (14)	−0.00176 (14)
Cl1	0.0209 (6)	0.0254 (6)	0.0236 (6)	0.0018 (4)	0.0048 (4)	−0.0043 (4)
N3	0.0149 (17)	0.013 (2)	0.0191 (18)	−0.0001 (16)	−0.0009 (13)	−0.0020 (17)
N1	0.015 (2)	0.014 (2)	0.021 (2)	0.0024 (16)	−0.0019 (16)	−0.0009 (17)
N2	0.014 (2)	0.020 (2)	0.021 (2)	0.0021 (15)	−0.0009 (15)	0.0000 (17)
C1	0.013 (2)	0.013 (2)	0.021 (2)	0.0036 (18)	0.0033 (19)	−0.003 (2)
C7	0.015 (2)	0.022 (3)	0.014 (2)	0.003 (2)	0.0024 (19)	−0.003 (2)
C14	0.014 (2)	0.014 (3)	0.018 (2)	0.004 (2)	0.0045 (16)	0.002 (2)
C11	0.021 (2)	0.028 (3)	0.015 (2)	0.003 (3)	−0.0064 (17)	−0.004 (3)
C6	0.034 (3)	0.016 (3)	0.023 (2)	0.000 (2)	0.000 (2)	−0.0004 (19)
C13	0.016 (3)	0.016 (3)	0.032 (3)	0.000 (2)	0.006 (2)	0.003 (2)
C12	0.019 (3)	0.018 (3)	0.030 (3)	−0.001 (2)	−0.0046 (19)	−0.001 (2)
C2	0.018 (2)	0.019 (3)	0.017 (2)	0.0019 (19)	−0.0027 (19)	−0.0001 (17)
C5	0.026 (3)	0.020 (3)	0.033 (3)	−0.002 (2)	0.007 (2)	0.002 (2)
C8	0.016 (3)	0.016 (2)	0.021 (2)	0.0029 (19)	0.0001 (18)	−0.0018 (18)
C4	0.021 (3)	0.012 (2)	0.018 (2)	−0.002 (2)	−0.001 (2)	0.0037 (17)
C10	0.020 (3)	0.021 (3)	0.011 (2)	0.005 (2)	−0.0014 (19)	−0.001 (2)
C9	0.020 (3)	0.021 (3)	0.020 (3)	0.003 (2)	0.001 (2)	0.001 (2)
C3	0.024 (3)	0.013 (3)	0.033 (3)	−0.004 (2)	0.003 (2)	−0.001 (2)
Br1	0.0209 (6)	0.0254 (6)	0.0236 (6)	0.0018 (4)	0.0048 (4)	−0.0043 (4)

### Geometric parameters (Å, º)

**Table:**

Rh1—Cl1	2.4308 (11)	C6—H6C	0.9800
Rh1—C1	2.016 (5)	C6—C4	1.521 (6)
Rh1—C7	2.125 (5)	C13—H13A	0.9900
Rh1—C14	2.121 (4)	C13—H13B	0.9900
Rh1—C11	2.242 (5)	C13—C12	1.528 (7)
Rh1—C10	2.221 (5)	C12—H12A	0.9900
Rh1—Br1	2.4308 (11)	C12—H12B	0.9900
N3—C1	1.359 (6)	C2—H2	0.9500
N3—C2	1.376 (6)	C5—H5A	0.9800
N3—C4	1.492 (6)	C5—H5B	0.9800
N1—N2	1.390 (5)	C5—H5C	0.9800
N1—C1	1.347 (6)	C5—C4	1.519 (7)
N1—C3	1.458 (6)	C8—H8A	0.9900
N2—C2	1.301 (6)	C8—H8B	0.9900
C7—H7	1.0000	C8—C9	1.538 (6)
C7—C14	1.415 (8)	C4—H4	1.0000
C7—C8	1.530 (7)	C10—H10	1.0000
C14—H14	1.0000	C10—C9	1.517 (7)
C14—C13	1.508 (7)	C9—H9A	0.9900
C11—H11	1.0000	C9—H9B	0.9900
C11—C12	1.527 (7)	C3—H3A	0.9800
C11—C10	1.371 (8)	C3—H3B	0.9800
C6—H6A	0.9800	C3—H3C	0.9800
C6—H6B	0.9800		
			
C1—Rh1—Cl1	87.93 (14)	C4—C6—H6C	109.5
C1—Rh1—C7	92.47 (19)	C14—C13—H13A	108.9
C1—Rh1—C14	88.55 (19)	C14—C13—H13B	108.9
C1—Rh1—C11	161.7 (2)	C14—C13—C12	113.4 (4)
C1—Rh1—C10	162.22 (19)	H13A—C13—H13B	107.7
C1—Rh1—Br1	87.93 (14)	C12—C13—H13A	108.9
C7—Rh1—Cl1	158.76 (15)	C12—C13—H13B	108.9
C7—Rh1—C11	89.1 (2)	C11—C12—C13	112.0 (4)
C7—Rh1—C10	82.02 (19)	C11—C12—H12A	109.2
C7—Rh1—Br1	158.76 (15)	C11—C12—H12B	109.2
C14—Rh1—Cl1	162.14 (16)	C13—C12—H12A	109.2
C14—Rh1—C7	38.9 (2)	C13—C12—H12B	109.2
C14—Rh1—C11	81.30 (16)	H12A—C12—H12B	107.9
C14—Rh1—C10	97.40 (18)	N3—C2—H2	124.1
C14—Rh1—Br1	162.14 (16)	N2—C2—N3	111.7 (4)
C11—Rh1—Cl1	97.08 (13)	N2—C2—H2	124.1
C11—Rh1—Br1	97.08 (13)	H5A—C5—H5B	109.5
C10—Rh1—Cl1	91.19 (13)	H5A—C5—H5C	109.5
C10—Rh1—C11	35.8 (2)	H5B—C5—H5C	109.5
C10—Rh1—Br1	91.19 (13)	C4—C5—H5A	109.5
C1—N3—C2	108.6 (4)	C4—C5—H5B	109.5
C1—N3—C4	124.6 (4)	C4—C5—H5C	109.5
C2—N3—C4	126.8 (4)	C7—C8—H8A	108.7
N2—N1—C3	119.4 (4)	C7—C8—H8B	108.7
C1—N1—N2	113.8 (4)	C7—C8—C9	114.3 (4)
C1—N1—C3	126.9 (4)	H8A—C8—H8B	107.6
C2—N2—N1	102.8 (4)	C9—C8—H8A	108.7
N3—C1—Rh1	128.5 (3)	C9—C8—H8B	108.7
N1—C1—Rh1	128.4 (3)	N3—C4—C6	109.8 (4)
N1—C1—N3	103.1 (4)	N3—C4—C5	110.2 (4)
Rh1—C7—H7	113.5	N3—C4—H4	108.0
C14—C7—Rh1	70.4 (3)	C6—C4—H4	108.0
C14—C7—H7	113.5	C5—C4—C6	112.6 (4)
C14—C7—C8	125.4 (4)	C5—C4—H4	108.0
C8—C7—Rh1	112.8 (3)	Rh1—C10—H10	114.0
C8—C7—H7	113.5	C11—C10—Rh1	72.9 (3)
Rh1—C14—H14	113.8	C11—C10—H10	114.0
C7—C14—Rh1	70.7 (3)	C11—C10—C9	126.1 (4)
C7—C14—H14	113.8	C9—C10—Rh1	107.7 (3)
C7—C14—C13	126.5 (5)	C9—C10—H10	114.0
C13—C14—Rh1	109.9 (3)	C8—C9—H9A	108.9
C13—C14—H14	113.8	C8—C9—H9B	108.9
Rh1—C11—H11	114.6	C10—C9—C8	113.2 (4)
C12—C11—Rh1	110.1 (3)	C10—C9—H9A	108.9
C12—C11—H11	114.6	C10—C9—H9B	108.9
C10—C11—Rh1	71.3 (3)	H9A—C9—H9B	107.7
C10—C11—H11	114.6	N1—C3—H3A	109.5
C10—C11—C12	123.6 (5)	N1—C3—H3B	109.5
H6A—C6—H6B	109.5	N1—C3—H3C	109.5
H6A—C6—H6C	109.5	H3A—C3—H3B	109.5
H6B—C6—H6C	109.5	H3A—C3—H3C	109.5
C4—C6—H6A	109.5	H3B—C3—H3C	109.5
C4—C6—H6B	109.5		
			
Rh1—C7—C14—C13	−100.9 (4)	C11—C10—C9—C8	46.9 (6)
Rh1—C7—C8—C9	−8.0 (5)	C12—C11—C10—Rh1	102.2 (4)
Rh1—C14—C13—C12	−40.3 (5)	C12—C11—C10—C9	2.4 (7)
Rh1—C11—C12—C13	−16.0 (6)	C2—N3—C1—Rh1	−178.6 (3)
Rh1—C11—C10—C9	−99.8 (5)	C2—N3—C1—N1	0.9 (5)
Rh1—C10—C9—C8	−34.5 (5)	C2—N3—C4—C6	−50.2 (6)
N1—N2—C2—N3	0.2 (5)	C2—N3—C4—C5	74.5 (5)
N2—N1—C1—Rh1	178.7 (3)	C8—C7—C14—Rh1	104.6 (4)
N2—N1—C1—N3	−0.8 (5)	C8—C7—C14—C13	3.7 (7)
C1—N3—C2—N2	−0.8 (5)	C4—N3—C1—Rh1	1.1 (6)
C1—N3—C4—C6	130.1 (5)	C4—N3—C1—N1	−179.4 (4)
C1—N3—C4—C5	−105.2 (5)	C4—N3—C2—N2	179.5 (4)
C1—N1—N2—C2	0.4 (5)	C10—C11—C12—C13	−96.4 (6)
C7—C14—C13—C12	39.9 (7)	C3—N1—N2—C2	179.3 (4)
C7—C8—C9—C10	29.2 (6)	C3—N1—C1—Rh1	−0.1 (7)
C14—C7—C8—C9	−89.4 (6)	C3—N1—C1—N3	−179.7 (4)
C14—C13—C12—C11	37.3 (6)		
